# Rapid Cycle Deliberate Practice: Application to Neonatal Resuscitation

**DOI:** 10.15766/mep_2374-8265.10534

**Published:** 2017-01-30

**Authors:** Karen Patricia, Jennifer Arnold, Daniel Lemke

**Affiliations:** 1 Assistant Professor of Pediatrics and Neonatology, Baylor College of Medicine; 2 Assistant Professor of Pediatrics-Emergency Medicine, Baylor College of Medicine

**Keywords:** Simulation, Neonatal Resuscitation, Rapid Cycle Deliberate Practice, RCDP, NRP

## Abstract

**Introduction:**

This curriculum includes two simulation cases for neonatal resuscitation training using the rapid cycle deliberate practice (RCDP) technique. RCDP is a simulation-based curriculum that presents participants with rounds of increasing difficulty in rapid repetition, interspersing brief, direct feedback within the simulation. In contrast, traditional debriefing focuses on learning after the scenario is complete. Traditional debriefing usually utilizes advocacy-inquiry debriefing but allows less opportunity for practice.

**Methods:**

Each case provides a neonatal resuscitation scenario (respiratory failure secondary to perinatal compromise and cardiac arrest secondary to placental abruption) for a term newborn in the delivery room. The curriculum utilizes high-fidelity neonatal mannequins with learner teams of three to six multidisciplinary teammates who participate in scenarios of increasing difficulty to revive a neonate. Learners can include a spectrum from beginning to advanced neonatal resuscitation providers. Learners are expected to perform the appropriate steps per the neonatal resuscitation program algorithm in addition to exhibiting effective crisis resource management skills.

**Results:**

Immediate assessment of learner performance and feedback within the RCDP model is more directive, which allows for rapid resumption of practice. The instructor may also choose to pause and back up or to pause and restart, depending on the correction needed.

**Discussion:**

Neonatal resuscitation program teaching utilizes a neonatal resuscitation performance evaluation, which may be used to guide opportunities for feedback within RCDP.

## Educational Objectives

By the end of this session, the learner will be able to:

1. Perform effectively as a team member during neonatal resuscitation with fetal depression and placental abruption.
2. Perform key steps of resuscitation within defined times and in correct order as outlined by the neonatal resuscitation program.
3. Demonstrate key team communication skills such as sharing of mental models, closed-loop communication, and clear role definition throughout the simulation scenarios.

## Introduction

Simulation-based medical education has become the standard technique for neonatal resuscitation programs (NRPs).^1^ Simulation-based medical education utilizes a theory of deliberate practice.^2^ Deliberate practice was first described by K. Anders Ericsson, an educational psychologist, as a framework for mastery learning. This theory of learning involves motivated learners, well-defined learning objectives, and focused and repetitive practice incorporating precise measurements of performance and informative feedback.^3–5^ By having learners repeat multiple scenarios with high-fidelity simulation, the goal is that there will be an improvement in advanced resuscitation skills.^2,4^

Elizabeth A. Hunt of Johns Hopkins University expanded on Ericsson's idea of deliberate practice and coined the phrase *rapid cycle deliberate practice* (RCDP). The goal of this training method is to spend more time in practice and less time in debriefing. The instructor provides immediate and specific feedback to learners during practice cycles. The initial cycle focuses on basic skills, and each cycle has an individual goal as well as being part of an overall progression through the entire case scenario. Learners repeat the same skills from cycle to cycle, with each cycle increasing in difficulty. The instructor may stop scenarios to provide feedback and then have the teams restart. The focus is on teaching with repetition in a time-sensitive situation as opposed to giving verbal feedback after a simulation, as in the traditional debriefing method. Hunt and colleagues recently published the first study looking at RCDP in pediatric cardiac arrest education.^7^ There, the implementation of the training program resulted in an associated improvement in performance by pediatric residents during simulated cardiac arrests when measuring time to chest compressions and time to defibrillation.^7^ Within the pediatric resuscitation curriculum, educational scenarios for pulseless electrical activity and ventricular tachycardia have also been published on MedEdPORTAL.^8^

RCDP is an innovative method that has been shown to be effective in pediatric resuscitation. Through practice, we have found that RCDP is best applied in simulation situations involving algorithms. NRP is an algorithmic strategy for resuscitation very similar to previous methods that RCDP has been applied to. The goal for the development of this curriculum was to utilize its cases in an upcoming study evaluating the applicability of RCDP within NRP teaching. Based on NRP guidelines, the neonatal resuscitation performance evaluation (NRPE) is a validated scoring instrument for assessing team performance during NRP training scenarios. This tool has previously been modified to fit studies on NRP performance and training techniques. It allows for evaluation of preparation, communication, bag/mask ventilation, chest compressions, intubation, and medications as necessary for each scenario.^2^ We used the NRPE to determine the success of each trained group as well as to guide the goals of the initial case development.

## Methods

RCDP, the educational approach utilized in this simulation scenario (Appendix A), is a practice that trains to mastery and is best used for teaching choreographed time-sensitive interventions, such as code events, to teams. The repetitive nature of a training case allows for correction of mistakes while the continual practice encourages muscle memory and long-term memory development. The simulation case file (Appendix A) contains two simulation cases that utilize the breakdown technique of RCDP. Each case represents a neonatal delivery room resuscitation, with difficulty increasing as the rounds progress. The cases require learners to perform a full resuscitation, including the use of positive pressure ventilation, endotracheal intubation, chest compressions, emergent umbilical venous catheter placement, and administration of epinephrine.

We use a recreation of the delivery room as the environment for these scenarios. To the best of our ability, we visually reassemble the layout of current delivery rooms. Each simulation room is equipped with a warmer and a resuscitation cart that are the same as those used in the actual delivery rooms. A full list of necessary presimulation setup equipment is given in Appendix A.

A simulation-trained instructor leads the education and debriefing. Additional personnel include a technical support person, who operates the mannequin while the lead instructor focuses on the learners and the simulation scenario, and a simulated instructor, who acts as the labor and delivery nurse. The simulated instructor provides the patient history and situation upon the learner group's arrival in addition to assisting the group if it is not adequately staffed for a full resuscitation. Each learner group should include a multidisciplinary neonatal resuscitation team of three to six members. Roles that may be covered include team leader, airway, circulation, and, if available, access, medications, and recorder. If functioning within a smaller code team, some members may play more than one role.

For the assessment (Appendix B), the lead instructor uses a modified NRPE, ensuring that all steps have been completed. The modified NRPE serves as a guide for teaching points and helps identify any critical steps missed during the session when the instructor may stop for a teaching moment. This tool can be used throughout as a guide for the instructor. As the instructor gains confidence with the material, the assessment may be completed at the end of the session.

For RCDP, debriefing is done within the simulation scenario itself via a start-and-stop method of teaching in quick bursts and fixing components, thereby allowing practice to mastery by the simulated code team. For this curriculum, there are identified stopping points as well as optional stopping points based on learners' performance during the simulation. The actual debriefing can lead to stops with continuation, with backing up 30–60 seconds, and with a full restart. Within the debriefing, it is essential for the instructor to provide praise along with the directive feedback. Tips for teaching within the RCDP method are found in the debriefing materials (Appendix C).

## Results

Since the finalization of this NRP RCDP curriculum, we have taught 128 individuals in 25 groups of multidisciplinary teams. Each team has consisted of four to six learners, including physicians (attendings, fellows, and residents), nurses, advanced practice providers, and respiratory therapists. All learners have expressed very high levels of satisfaction with the RCDP teaching methodology. Currently, a randomized controlled trial comparing the RCDP scenarios with traditional scenarios, which ended in the fall of 2015, is undergoing video review completion.

All of the instructors who participated in the course had completed a 2-day simulation instructor course as well as receiving NRP instructor certification. They were recruited on a voluntary basis and underwent further specialized training in the RCDP model. Over three afternoon training sessions of 3–4 hours apiece, each trainee was able to participate in the scenarios as well as be the instructor debriefed by an RCDP expert during the practice session. The goal was to ensure the trainees were comfortable with the repetitive nature and short directive actions of RCDP feedback, as opposed to the more open-ended questioning of traditional feedback. For this curriculum, our team of instructors consisted of a combination of two neonatal attendings, three neonatal nurse practitioners, and a senior nurse in the neonatal department. They brought a wealth of clinical information and experience to the overall training program.

The majority of learners provided very positive comments about the RCDP training method. In general, learners commented that RCDP helped them gain confidence, provided immediate feedback, was helpful with critical thinking, and offered positive feedback. Specific qualitative comments from learners included the following:

• “The rapid cycle was great—the most I've gotten out of any sim.”
• “RCDP—liked it better than traditional—gained more confidence that way.”
• “I think the rapid sequence technique is more helpful because it provided immediate feedback and what we can improve upon.”
• “The new method really reflected after the final simulation in which I felt we performed wonderfully as a team.”

Constructive criticism regarding the simulation, noting that it was, among other things, very tiring and difficult to start over, included the following:

• “Hard to start back over on the rapid training but effective in learning things faster.”
• “Rapid cycle was very overwhelming initially with all the frequent pauses; towards the end I could see how rapid simulation helped us work better as a team.”

## Discussion

Initially, we started by adapting current NRP cases to work within the RCDP technique. To do this, we needed to create rounds that provided participants with adequate repetition of basics and new challenges with each round. We then tested these cases on master learners (attending and fellow neonatologists) who were experts with NRP. In these sessions, we worked out specific content for each of the cases and the best-practice choreography for role clarity of the code event. One of the biggest challenges we encountered was the varying opinions of the master learners and how to best determine an organized choreography and work flow for a code event. RCDP is best used with scenarios that require a team of providers to follow a clearly defined path under time pressure. This requirement describes the NRP. However, officially defined roles and choreography for implementation of the interventions within the NRP algorithm do not yet exist, so we had to create our own recommendations for roles and positioning during the training sessions. We made these recommendations based on expert opinion from the master learners during our pilot RCDP simulation sessions. One future direction for this work would be to further evaluate the optimal roles and positions for implementing the NRP algorithm during a neonatal resuscitation.

A challenge we encountered while training instructors was transitioning them from the traditional methodology to RCDP. Our instructors had been previously trained in traditional postsimulation debriefing. Traditional debriefing utilizes probing and open-ended questions, whereas RCDP uses clear and succinct recommendations. RCDP can incorporate traditional methods of debriefing if the learners continually miss a concept. As per RCDP, practice makes perfect even in the teaching of instructors: We found that the more they practiced, the more comfortable they became with the model. We also found that briefly reviewing the goals of each cycle as well as the RCDP teaching tips as a refresher before every teaching session was helpful. These miniature review sessions became less necessary after several rounds of teaching. We recommend that before teaching the sessions, the instructors all practice together, acting as both the learners and the instructors, to fully understand the flow of the cases.

Actual learners reported thoroughly enjoying the entire RCDP educational session and creating bonds with their teammates in a short time period during the simulations. Learners who had taken NRP in the past also reported that the ability to practice repetitively enhanced their learning compared to traditional postsimulation debriefing. The session was so well received that many learners have now requested RCDP simulation-based medical education over traditional debriefing. Lastly, a future direction for evaluation of this curriculum will be to delve into studying the retention time of teaching with this method, which will be useful in determining its true efficacy.

## Appendices

A. Simulation Case.docx
B. Critical Actions.docx
C. Debriefing Materials.docx All appendices are peer reviewed as integral parts of the Original Publication.
