# Supplementary material for: Rapid Cycle Deliberate Practice: Application to Neonatal Resuscitation
Source: MedEdPORTAL. 2017 Jan 30;13:10534. doi: 10.15766/mep_2374-8265.10534 (PMC6342166; doi:10.15766/mep_2374-8265.10534)
Supplement: Supplementary file 1 — A. Simulation Case.docx B. Critical Actions.docx C. Debriefing Materials.docx [file mep-13-10534-s001.zip › A. Simulation Case.docx]

| **Appendix A: MedEdPORTAL Simulation Case 1**  **SIMULATION CASE TITLE: Neonatal Resuscitation – PPV and RCDP**  **AUTHORS: Karen Patricia, MD** | |
| --- | --- |
| **PATIENT NAME: Baby G**  **PATIENT AGE: Newborn**  **CHIEF COMPLAINT: Delivery of a term newborn with fetal decelerations** | |
|  | |
| **Brief narrative description of case** | You are attending the delivery of a full term newborn with fetal decelerations. The infant will be placed on the warmer requiring resuscitation. The case will consist of three rounds of learning with increasing difficulty in each added scenario. The overall goal is for the learner to master the initial steps of infant resuscitation through intubation, according to standard neonatal resuscitation guidelines. |
| **Primary Learning Objectives:** | All Rounds:   1. Team members will verbalize using closed loop communication for all tasks. 2. Team members will demonstrate respect to each other through language and behavior. 3. Team leader will vocalize their role as well as all team members assigned roles by the start of the resuscitation.   Round 1:   1. Team will demonstrate proper equipment preparation and recognition of newborn in distress. 2. A member of the team will correctly demonstrate initial NRP step of warm, dry and stimulation. 3. Team leader will be able to vocalize the shared mental model of next steps, including possible need for PPV.   Round 2 (objectives for round 1 and additionally):   1. Team members will identify heart rate <100 and need for positive pressure ventilation. 2. Team will effectively provide positive pressure ventilation using correct technique   Round 3 (all above objectives and additionally):   1. Team members will identify heart rate <60 and provide PPV. 2. Team members will correctly identify ineffective ventilation, utilizing the mnemonic MRSOPA, and demonstrate intubation. |
| **Critical Actions** | - Identify neonate in need of resuscitation - Have all equipment available to resuscitate infant - Provide effective PPV - Provide effective trouble-shooting of ineffective ventilation - Successful intubation |
| **Learner Preparation** | The learners should have studied the NRP algorithm and either be trained in completing the algorithm and practicing scenarios or be training for the completion of the NRP class. The simulations are ineffective if the algorithm has not already been studied. |

| Initial Presentation | | | |
| --- | --- | --- | --- |
| **Initial vital signs** | Round 1:  HR 70 bpm, RR 4, SpO2 61%, Tone Limp, Color blue, Crying: None, BS: Coarse  Round 2:  HR 70 bpm, RR 0, SpO2 50%, Tone Limp, Color blue, Crying: None, BS: None - apneic  Round 3:  HR 40 bpm, RR 0, SpO2 40%, Tone Limp, Color blue, Crying: None, BS: None – apneic, obstruction of airway on to simulate difficult respirations | | |
| **Overall Appearance** | Patient is not born when learners enter the room. Delivery room set-up with warmer available. Neo code cart present and patient covered with a blanket | | |
| **Simulation Room Equipment** | Warmer  Resuscitation Cart (similar to cart used for your L&D room)  SimNewB (high fidelity mannequin, Laerdal) or other high fidelity neonatal mannequin  Warm blankets  Umbilical venous line set up  Neonatal crash cart with emergency medications  Infant warming bed with Apgar timer  Bulb suction  Neonatal stethoscope  Laryngoscopes with blades (00, 0, 1)  Endotracheal tubes (ETT) with stylets (sizes 2.5, 3.0, 3.5 and 4.0)  Self-inflating neonatal resuscitation bag with pressure release valve  Flow-inflating neonatal resuscitation bag  Neonatal face masks (preemie, newborn, and infant)  Cardiac monitor w/ pulse oximetry  CO_2_ detectors  Oral Gastric catheters  Neonatal suction catheters  Wall suction  Tape or device to secure ETT  Gloves, surgical hats, masks, and gowns  Simulated blood for umbilicus. | | |
| **Actors and roles in the room at case start** | L&D Baby nurse, their role is to provide initial reason as to why the neonatal resuscitation team was activated and maternal history. Also available to the team after baby born to update parents/make phone calls.  Role played by the debriefer during Rapid Cycle Cases. May also be played by extra staff member available to act. Does not require trained standardized patient. | | |
| **HPI** | “You are attending the delivery of a full term newborn with fetal decelerations. There are no concerning historical data for this patient.”  Round 1: “You have one minute to prepare your equipment (baby delivered and placed on warmer at one minute).”  Round 2/3: Start with equipment ready, HPI stem given with immediate placement of baby on warmer.  Additional history if the team requests:  Mom is a 25 yo G1P0, 39 weeks gestation, early prenatal care with normal history and normal serologies. Clear Fluid. | | |
| **Past Medical/Surgical History** | **Medications** | **Allergies** | **Family History** |
| Negative maternal past history | Maternal: prenatal vitamins | NKDA | No significant family history |
| **Initial Physical Examination** | | | |
| **General** | Low tone, not crying, not moving | | |
| **HEENT** | No dysmorphic features | | |
| **Neck** |  | | |
| **Lungs** | Round 1: crackles Round 2/3: apneic, no breath sounds | | |
| **Cardiovascular** | bradycardia | | |
| **Abdomen** | Soft, non-distended | | |
| **Neurological** | No tone | | |
| **Skin** | Color blue | | |
| **GU** | female | | |
| **Psychiatric** | n/a | | |

| Instructor Notes - Changes and CASE Branch Points  This section should be a list with detailed description of each step than may happen during the case. If medications are given, what is the response? Do changes occur at certain time points? Should the nurse or other participant prompt the learners at given points? Should new actors or participants enter, and when? Are there specific things the patient will say or do at given times? There are a few examples given, but it is expected that most cases will have many more changes and potential branch points.. | | |
| --- | --- | --- |
| **Intervention / Time point** | **Change in Case** | **Additional Information** |
| Identification of roles | Establishment of good communication, prior to delivery of newborn | Team leader assigns: Airway, Circulation, Access Roles |
| Preparation of Equipment |  | Airway: Sets up blade, ET tubes (under mattress, away from heat), Bulb suction  Circulation: Sets up bag, suction, oxygen (21% for term infant), pulse ox ready, CO2 detector  Access: UVC kit and PIV kit ready/available, Hat, temp probe and bed on – ready for APGAR timer |
| **Round 1:** |  |  |
| Infant is born |  | APGAR timer started  Assessment of breathing, tone verbalized  Warm, Dry, Stimulate  Remove wet blankets |
| 30 seconds in to resuscitation | Patient HR increasing to 140, RR 44, SpO2 trending up, crying | Team continues to warm, dry, stimulate baby |
|  | O2 sats continue to improve | Team leader shares mental model of need for CPAP/PPV but that no further intervention should be needed as patient is improving |
| **Round 2** |  | Begins when all Round 1 objectives are met |
| Infant is born |  | APGAR timer started  Assessment of breathing, tone verbalized  Warm, Dry, Stimulate  Remove wet blankets |
| 30 seconds in to resuscitation | No clinical change to patient yet | Pulse Check – recognize HR <100 and apnea  Airway Role: State “The HR is <100, I am starting PPV.” |
| 30 sec to 1 minute of resuscitation |  | Effective Ventilations given – insufflations over 0.5 seconds, occurring every 1-2 seconds, counted aloud as “Breath Two Three” |
| 1 minute | HR improves to 140, SpO2 improves to 85, RR to 40 | SpO2 monitor placed on Right hand |
|  | SpO2 continues to improve | Team leader shares mental model of potential need to check ventilation (using MRSOPA) but that no further intervention should be needed as patient is improving with PPV |
| **Round 3** |  | Begins when all Round 2 objectives are met |
| Infant is born |  | APGAR timer started  Assessment of breathing, tone verbalized  Warm, Dry, Stimulate  Remove wet blankets |
| 30 seconds in to resuscitation | No clinical change to patient | Pulse Check – recognize HR <100 and apnea  Airway Role: State “The HR is <100, I am starting PPV.” |
| 30 sec to 1 minute of resuscitation |  | Non-effective ventilations; no chest rise seen (mannequin obstructed airway) |
| 1 minute | HR improves to 80, SpO2 40, RR 0 | Recognized continued apnea with difficulty providing respirations, yet HR >60 |
| 1 minute to 1:30 seconds |  | MR.SOPA – mask adjustment, reposition head, secretions, adjust oxygen, pressure, alternate airway (in sequential order – starting with MR, no improvement followed by each individual action with no improvement)  - Intubation materials readied |
| 1:30 | No change to patient | Patient is intubated using correct technique:  -Stabilize head in sniffing position  -Oxygenation before attempt with free-flowing oxygen  -Lift Laryngoscope up instead of rocking – slide right to left to sweep tongue  -Take less than 30 seconds or re-oxygenate patient  -Hold tube firmly against palate while removing blade  - Assess airway after with CO2 monitor, chest rise, auscultation, HR Improvement |
| 2 minutes | HR 140, RR 44 (rate of ventilation 40-60), SpO2 70% and relieve airway obstruction | Team leader provides a shared mental model of events and verbalizes that patient will need admission to NICU |

**Ideal Scenario Flow**

Provide a detailed narrative description of the way this case should flow if participants perform in the ideal fashion.

The purpose of RCDP is to practice until perfection of each round given:

For round 1 the ideal scenario is that the team recognizes that a neonatal resuscitation is about to occur. They prepare all equipment necessary for resuscitation including: airway and access supplies prior to infant being delivered. Upon delivery, team recognizes a limp, not crying infant and begins to warm, dry and stimulate the patient. This is effective and the patient responds with improved tone, heart rate, spontaneous respirations and crying.

For round 2 the ideal scenario is that the team recognizes that a neonatal resuscitation is about to occur. They prepare all equipment necessary for resuscitation including: airway and access supplies prior to infant being delivered. Upon delivery, team recognizes a limp, not crying infant and begins to warm, dry and stimulate the patient. There is no response and the team provides effective positive pressure ventilation. With effective ventilation the patient responds with improved tone, heart rate, spontaneous respirations and crying.

For round 3 the ideal scenario is that the team recognizes that a neonatal resuscitation is about to occur. They prepare all equipment necessary for resuscitation including: airway and access supplies prior to infant being delivered. Upon delivery, team recognizes a limp, not crying infant and begins to warm, dry and stimulate the patient. There is no response and the team provides positive pressure ventilation. The positive pressure ventilation is not effective with no chest rise or improvement in vital signs. The team then trouble shoots with ventilation utilizing MR. SOPA with no improvement and intubation is attempted. Upon successful ventilation with successful intubation the patient responds to the effective ventilation and has improved tone, heart rate, spontaneous respirations and crying.

**Anticipated Management Mistakes**

1. Failure to set-up all equipment necessary: We found that when initiating the case, teams initially were not prepared with all equipment needed and unable to locate in procedure carts. We practiced and incorporated orientation to the equipment and cart.
2. Failure to check for successful intubation: Some of our learners were unable to successfully intubate despite prior practice on mannequin heads. Modified orientation to include practice times for advanced practitioners in the team to practice intubation on Sim Baby. Also allowed mannequin to remain with low Heart Rate until corrective measures or a re-attempt completed.
3. Failure to assign roles: We found that when running these simulations using RCDP, some of our learners were not accustomed to clearly defining roles and positions. They often just went to typical positions and tasks based on their current practice. During RCDP, there is an opportunity to get feedback on clearly defined roles and actual locations around the bed more closely given multiple opportunities to repeat this important teamwork skill. We modified our debriefing points to include an emphasis on assigning roles and positions during each neonatal resuscitation simulation.
4. Failure to use MR. SOPA in round 3: We found that in round 3 when ventilations were not effective, most teams were likely to prepare for intubation instead of completing MR. SOPA. In this situation we allowed the mannequin to remain with depressed vital signs after intubation to prompt them for the need to evaluate airway and ventilations more closely.

| **Appendix A: MedEdPORTAL Simulation Case 2**  **SIMULATION CASE TITLE: Neonatal Resuscitation – Placental abruption and RCDP**  **AUTHORS: Karen Patricia, MD** | |
| --- | --- |
| **PATIENT NAME: Baby D**  **PATIENT AGE: Newborn**  **CHIEF COMPLAINT: Delivery of a term newborn complicated by placenta previa** | |
|  | |
| **Brief narrative description of case** | You are attending the delivery of a full term newborn with known maternal placenta previa. The infant will be placed on the warmer requiring resuscitation. The case will consist of four rounds of learning with increasing difficulty in each added scenario. The overall goal is for the learner to master the steps of infant resuscitation through a megacode, including intubation, chest compressions, line placement and epinephrine. |
| **Primary Learning Objectives** | All Rounds:   1. Team members will verbalize using closed loop communication for all tasks. 2. Team members will demonstrate respect to each other through language and behavior. 3. Team leader will vocalize their role as well as all team members assigned roles by the start of the resuscitation.   Round 1:   1. Team will demonstrate proper equipment preparation and recognition of newborn in distress. 2. A member of the team will correctly demonstrate initial NRP step of warm, dry and stimulation. 3. Team leader will be able to vocalize the shared mental model of next steps, including possible need for PPV. 4. Team will effectively provide positive pressure ventilation using correct technique   Round 2 (objectives for round 1 and additionally):   1. Team members will correctly identify ineffective ventilation, utilizing the mnemonic MR.SOPA, and demonstrate intubation.   Round 3 (all above objectives and additionally):   1. Team members will identify heart rate <60 and start chest compressions. 2. Team members will correctly provide effective chest compressions with correct ventilation ration 3:1   Round 4 (all above objectives and additionally):   1. Team will identify need for vascular access and circulation role will correctly place emergent umbilical venous catheter 2. Team correctly administers dose of IV epinephrine 3. Team requests for pRBCs (unit of O negative) from blood bank |
| **Critical Actions** | - Identify neonate in need of resuscitation - Have all equipment available to resuscitate infant - Provide effective PPV - Provide effective trouble-shooting of ineffective ventilation - Successful intubation - Initiation of chest compressions - Placement of UVC - Administration of Epinephrine - Recognition of need for Volume, pRBCs |
| **Learner Preparation** | The learners should have studied the NRP algorithm and either be trained in completing the algorithm and practicing scenarios or be training for the completion of the NRP class. The simulations are ineffective if the algorithm has not already been studied. |

| Initial Presentation | | | |
| --- | --- | --- | --- |
| **Initial vital signs** | Round 1:  HR 70 bpm, RR 4, SpO2 60%, Tone Limp, Color blue, Crying: None, BS: gasping  Round 2:  HR 70 bpm, RR 0, SpO2 50%, Tone Limp, Color blue, Crying: None, BS: None - apneic  Round 3&4:  HR 40 bpm, RR 0, SpO2 40%, Tone Limp, Color blue, Crying: None, BS: None – apneic, obstruction of airway on to simulate difficult respirations | | |
| **Overall Appearance** | Patient is not born when learners enter the room. Delivery room set-up with warmer available. Neo code cart present and patient covered with a blanket Upon visualization of baby, they will find a moulaged with blood newborn. | | |
| **Simulation Room Equipment** | Warmer  Resuscitation Cart (similar to cart used for your L&D room)  SimNewB (high fidelity mannequin, Laerdal) or other high fidelity neonatal mannequin  Warm blankets  Umbilical venous line set up  Neonatal crash cart with emergency medications  Infant warming bed with Apgar timer  Bulb suction  Neonatal stethoscope  Laryngoscopes with blades (00, 0, 1)  Endotracheal tubes (ETT) with stylets (sizes 2.5, 3.0, 3.5 and 4.0)  Self-inflating neonatal resuscitation bag with pressure release valve  Flow-inflating neonatal resuscitation bag  Neonatal face masks (preemie, newborn, and infant)  Cardiac monitor w/ pulse oximetry  CO_2_ detectors  Oral Gastric catheters  Neonatal suction catheters  Wall suction  Tape or device to secure ETT  Gloves, surgical hats, masks, and gowns  Simulated blood for umbilicus. | | |
| **Actors and roles in the room at case start** | L&D Baby nurse, their role is to provide initial reason as to why the neonatal resuscitation team was activated and maternal history. Also available to the team after baby born to update parents/make phone calls.  Role played by the debriefer during Rapid Cycle Cases. May also be played by extra staff member available to act. Does not require trained standardized patient. | | |
| **HPI** | “You are attending the delivery of a full term newborn with known placenta previa. Patient is contracting and having vaginal bleeding. Decision made to proceed with an emergency C-section. There are no other pregnancy complications for this patient.  Round 1: “You have one minute to prepare your equipment (baby delivered and placed on warmer at one minute).”  Round 2/3: Start with equipment ready, HPI stem given with immediate placement of baby on warmer.  Additional history if the team requests:  Mom is a 28 yo G1P0, 39 weeks gestation, early prenatal care with normal history and normal serologies. Clear Fluid. | | |
| **Past Medical/Surgical History** | **Medications** | **Allergies** | **Family History** |
| Negative maternal past history | Maternal: prenatal vitamins | NKDA | No significant family history |
| **Initial Physical Examination** | | | |
| **General** | Low tone, not crying, not moving, covered in blood | | |
| **HEENT** | No dysmorphic features | | |
| **Neck** |  | | |
| **Lungs** | Round 1: gasping Round 2/3/4: apneic, no breath sounds | | |
| **Cardiovascular** | bradycardia | | |
| **Abdomen** | Soft, non-distended | | |
| **Neurological** | No tone | | |
| **Skin** | Color blue | | |
| **GU** | female | | |
| **Psychiatric** | n/a | | |

| Instructor Notes - Changes and CASE Branch Points  This section should be a list with detailed description of each step than may happen during the case. If medications are given, what is the response? Do changes occur at certain time points? Should the nurse or other participant prompt the learners at given points? Should new actors or participants enter, and when? Are there specific things the patient will say or do at given times? There are a few examples given, but it is expected that most cases will have many more changes and potential branch points.. | | |
| --- | --- | --- |
| **Intervention / Time point** | **Change in Case** | **Additional Information** |
| Identification of roles | Establishment of good communication, prior to delivery of newborn | Team leader assigns: Airway, Circulation, Access Roles |
| Preparation of Equipment |  | Airway: Sets up blade, ET tubes (under mattress, away from heat), Bulb suction  Circulation: Sets up bag, suction, oxygen (21% for term infant), pulse ox ready, CO2 detector  Access: UVC kit and PIV kit ready/available, Hat, temp probe and bed on – ready for APGAR timer |
| **Round 1:** |  |  |
| Infant is born |  | APGAR timer started  Assessment of breathing, tone verbalized  Warm, Dry, Stimulate  Remove wet blankets |
| 30 seconds in to resuscitation | No clinical change to patient yet | Pulse Check – recognize HR <100 and apnea  Airway Role: State “The HR is <100, I am starting PPV.” |
| 30 sec to 1 minute of resuscitation |  | Effective Ventilations given – insufflations over 0.5 seconds, occurring every 1-2 seconds, counted aloud as “Breath Two Three” |
| 1 minute | HR improves to 140, SpO2 improves to 85, RR to 40 | SpO2 monitor placed on Right hand |
|  | SpO2 continues to improve | Team leader shares mental model of potential need to check ventilation (using MRSOPA) but that no further intervention should be needed as patient is improving with PPV |
| **Round 2** |  | Begins when all Round 1 objectives are met |
| Infant is born |  | APGAR timer started  Assessment of breathing, tone verbalized  Warm, Dry, Stimulate  Remove wet blankets |
| 30 seconds in to resuscitation | No clinical change to patient | Pulse Check – recognize HR <100 and apnea  Airway Role: State “The HR is <100, I am starting PPV.” |
| 30 sec to 1 minute of resuscitation |  | Non-effective ventilations; no chest rise seen (mannequin obstructed airway) |
| 1 minute | HR improves to 80, SpO2 40, RR 0 | Recognized continued apnea with difficulty providing respirations, yet HR >60 |
| 1 minute to 1:30 seconds |  | MR.SOPA – mask adjustment, reposition head, secretions, adjust oxygen, pressure, alternate airway (in sequential order – starting with MR, no improvement followed by each individual action with no improvement)  - Intubation materials readied |
| 1:30 | No change to patient | Patient is intubated using correct technique:  -Stabilize head in sniffing position  -Oxygenation before attempt with free-flowing oxygen  -Lift Laryngoscope up instead of rocking – slide right to left to sweep tongue  -Take less than 30 seconds or re-oxygenate patient  -Hold tube firmly against palate while removing blade  - Assess airway after with CO2 monitor, chest rise, auscultation, HR Improvement |
| 2 minutes | HR 140, RR 44 (rate of ventilation 40-60), SpO2 70% and relieve airway obstruction | Team leader provides a shared mental model of events and verbalizes that patient will need admission to NICU |
| **Round 3** |  | Begins when all Round 2 objectives are met |
| Infant is born |  | APGAR timer started  Assessment of breathing, tone verbalized  Warm, Dry, Stimulate  Remove wet blankets |
| 30 seconds in to resuscitation | No clinical change to patient | Pulse Check – recognize HR <100 and apnea  Airway Role: State “The HR is <100, I am starting PPV.” |
| 30 sec to 1 minute of resuscitation |  | Non-effective ventilations; no chest rise seen (mannequin obstructed airway) |
| 1 minute | HR improves to 80, SpO2 40, RR 0 | Recognized continued apnea with difficulty providing respirations, yet HR >60 |
| 1 minute to 1:30 seconds |  | MR.SOPA – mask adjustment, reposition head, secretions, adjust oxygen, pressure, alternate airway (in sequential order – starting with MR, no improvement followed by each individual action with no improvement)  - Intubation materials readied |
| 1:30 | No change to patient | Patient is intubated using correct technique:  -Stabilize head in sniffing position  -Oxygenation before attempt with free-flowing oxygen  -Lift Laryngoscope up instead of rocking – slide right to left to sweep tongue  -Take less than 30 seconds or re-oxygenate patient  -Hold tube firmly against palate while removing blade  - Assess airway after with CO2 monitor, chest rise, auscultation, HR Improvement |
| 1:30 to 2 minutes | No change to patient | -Successful intubation  -Circulation role: Recognize need to start chest compressions and state “The HR is <60, I am starting compressions at 3:1 ratio” |
| For 30 seconds |  | Continue chest compression with good ventilations via intubation |
|  | HR 140, RR 44, SpO2 70%, relieve airway obstruction |  |
| **Round 4** |  | Begins when all Round 3 objectives are met |
| Infant is born |  | APGAR timer started  Assessment of breathing, tone verbalized  Warm, Dry, Stimulate  Remove wet blankets |
| 30 seconds in to resuscitation | No clinical change to patient | Pulse Check – recognize HR <100 and apnea  Airway Role: State “The HR is <100, I am starting PPV.” |
| 30 sec to 1 minute of resuscitation |  | Non-effective ventilations; no chest rise seen (mannequin obstructed airway) |
| 1 minute | HR improves to 80, SpO2 40, RR 0 | Recognized continued apnea with difficulty providing respirations, yet HR >60 |
| 1 minute to 1:30 seconds |  | MR.SOPA – mask adjustment, reposition head, secretions, adjust oxygen, pressure, alternate airway (in sequential order – starting with MR, no improvement followed by each individual action with no improvement)  - Intubation materials readied |
| 1:30 | No change to patient | Patient is intubated using correct technique:  -Stabilize head in sniffing position  -Oxygenation before attempt with free-flowing oxygen  -Lift Laryngoscope up instead of rocking – slide right to left to sweep tongue  -Take less than 30 seconds or re-oxygenate patient  -Hold tube firmly against palate while removing blade  - Assess airway after with CO2 monitor, chest rise, auscultation, HR Improvement |
| 1:30 to 2 minutes | No change to patient | -Successful intubation  -Circulation role: Recognize need to start chest compressions and state “The HR is <60, I am starting compressions at 3:1 ratio” |
| 2 minutes | No improvement to patient | -Pulse check, HR <60  -Team may elect to administer ET epi (ET epinephrine dose 0.5-1 ml/kg. assume 3 kg for term baby) |
| 2:00 to |  | -Placement of UVC  -Administration of epinephrine (IV epinephrine dose 0.1-0.3 ml/kg. assume 3 kg for term baby)  -Administration of NS bolus (10 ml/kg/dose)  -Call for pRBCs (if not already recognized and done) |
| Every 30 seconds |  | Pulse check |
| After administration of epinephrine, bolus, pRBCs | HR 140, RR 44, SpO2 70%, relieve airway obstruction |  |

**Ideal Scenario Flow**

Provide a detailed narrative description of the way this case should flow if participants perform in the ideal fashion.

The purpose of RCDP is to practice until perfection of each round given:

For round 1 the ideal scenario is that the team recognizes that a neonatal resuscitation is about to occur. They prepare all equipment necessary for resuscitation including: airway and access supplies prior to infant being delivered. Upon delivery, team recognizes a limp, not crying infant and begins to warm, dry and stimulate the patient. There is no response and the team provides effective positive pressure ventilation. With effective ventilation the patient responds with improved tone, heart rate, spontaneous respirations and crying.

For round 2 the ideal scenario is that the team recognizes that a neonatal resuscitation is about to occur. They prepare all equipment necessary for resuscitation including: airway and access supplies prior to infant being delivered. Upon delivery, team recognizes a limp, not crying infant and begins to warm, dry and stimulate the patient. There is no response and the team provides positive pressure ventilation. The positive pressure ventilation is not effective with no chest rise or improvement in vital signs. The team then trouble shoots with ventilation utilizing MR. SOPA with no improvement and intubation is attempted. Upon successful ventilation with successful intubation the patient responds to the effective ventilation and has improved tone, heart rate, spontaneous respirations and crying.

For round 3 the ideal scenario is that the team recognizes that a neonatal resuscitation is about to occur. They prepare all equipment necessary for resuscitation including: airway and access supplies prior to infant being delivered. Upon delivery, team recognizes a limp, not crying infant and begins to warm, dry and stimulate the patient. There is no response and the team provides positive pressure ventilation. The positive pressure ventilation is not effective with no chest rise or improvement in vital signs. The team then trouble shoots with ventilation utilizing MR. SOPA with no improvement and intubation is attempted. The heart rate remains below 60 and the team initiates chest compressions. After 30 seconds of effective compressions and ventilation the patient responds and has improved tone and heart rate.

For round 4 the ideal scenario is that the team recognizes that a neonatal resuscitation is about to occur. They prepare all equipment necessary for resuscitation including: airway and access supplies prior to infant being delivered. Upon delivery, team recognizes a limp, not crying infant and begins to warm, dry and stimulate the patient. There is no response and the team provides positive pressure ventilation. The positive pressure ventilation is not effective with no chest rise or improvement in vital signs. The heart rate remains below 60 and the team initiates chest compressions. After a 30 second pulse check there is no improvement and the team successfully places an emergent umbilical venous catheter. Immediately IV epinephrine is given followed by a Normal Saline Bolus. The team has recognized the need for volume and requested pRBCs from the blood bank. After epinephrine and 2 rounds of volume the patients pulse returns.

**Anticipated Management Mistakes**

1. Failure to set-up all equipment necessary: We found that when initiating the case, teams initially were not prepared with all equipment needed and unable to locate in procedure carts. We practiced and incorporated orientation to the equipment and cart.
2. Failure to check for successful intubation: Some of our learners were unable to successfully intubate despite prior practice on mannequin heads. Modified orientation to include practice times for advanced practitioners in the team to practice intubation on Sim Baby. Also allowed mannequin to remain with low Heart Rate until corrective measures or a re-attempt completed
3. Failure to assign roles: We found that when running these simulations using RCDP, some of our learners were not accustomed to clearly defining roles and positions. They often just went to typical positions and tasks based on their current practice. During RCDP, there is an opportunity to get feedback on clearly defined roles and actual locations around the bed more closely given multiple opportunities to repeat this important teamwork skill. We modified our debriefing points to include an emphasis on assigning roles and positions during each neonatal resuscitation simulation.
4. Failure to recognize the need for volume resuscitation: We found that some learner teams would only provide epinephrine, but not volume during resuscitation. In order to help prompt learners for need of volume resuscitation in this situation, we allowed the mannequin to remain with a low heart rate even after epinephrine given to prompt need for volume.
