# Supplementary material for: Rapid Cycle Deliberate Practice: Application to Neonatal Resuscitation
Source: MedEdPORTAL. 2017 Jan 30;13:10534. doi: 10.15766/mep_2374-8265.10534 (PMC6342166; doi:10.15766/mep_2374-8265.10534)
Supplement: Supplementary file 1 — A. Simulation Case.docx B. Critical Actions.docx C. Debriefing Materials.docx [file mep-13-10534-s001.zip › B. Critical Actions.docx]

Modified Neonatal Resuscitation Program Evaluation

| **Learner Group (check box for type of group):** |  | **RCDP** |  | **Traditional** |
| --- | --- | --- | --- | --- |
| *total points only for YES answers* |  |  | Yes | No |
| **Prep Steps:** |  |  |  |  |
| Identify Team Roles |  |  |  |  |
| Check Warmer - on, heat on, blankets, hat |  |  |  |  |
| Check Bag ready |  |  |  |  |
| Check ET supplies available |  |  |  |  |
| Check IV supplies |  |  |  |  |
| UVC kit ready |  |  |  |  |
| Total Points: |  |  | /6 | |
| **Initial Steps** |  |  |  |  |
| Dried Infant |  |  |  |  |
| Positioned with neck slightly extended |  |  |  |  |
| Suctioned mouth then nose |  |  |  |  |
| Appropriate Tactile Stimulation |  |  |  |  |
| Total Points: |  |  | /4 | |
| **Communication of heart rate to Leader:** |  |  |  |  |
| HR checked by approved method |  |  |  |  |
| HR communicated (tapped w finger or verbal) |  |  |  |  |
| Total Points: |  |  | /2 | |
| **Bag/Mask Ventilation:** |  |  |  |  |
| Appropriate decision based on clinical condition: |  |  |  |  |
| Technique: |  |  |  |  |
| Correct Rate (40-60) |  |  |  |  |
| Correct Pressure and seal (adequate chest rise) |  |  |  |  |
| Applies MR. SOPA correctly |  |  |  |  |
| Re-eval for response (30 seconds HR check) |  |  |  |  |
| Total Points: |  |  | /5 | |
| **Chest** **Compression** |  |  |  |  |
| Appropriate decision based on clinical condition: |  |  |  |  |
| Technique: |  |  |  |  |
| Correct method (2 finger, or hands encircling chest) |  |  |  |  |
| Correct Rate (90 per minute) |  |  |  |  |
| Correct Ventilation Coordination (3:1) |  |  |  |  |
| Re-eval for response (30 seconds HR check) |  |  |  |  |
| Total Points: |  |  | /5 | |
| **Intubation**: |  |  |  |  |
| Appropriate decision based on clinical condition: |  |  |  |  |
| Technique: |  |  |  |  |
| Correct handling of laryngoscope |  |  |  |  |
| ETT position checked |  |  |  |  |
| Successful (<= 2 attempts Yes, >2 No) |  |  |  |  |
| Total Points: |  |  | /4 | |
| **Medications:** |  |  |  |  |
| Appropriate Use of Epinephrine |  |  |  |  |
| Dose and Route |  |  |  |  |
| Re-evaluated for response |  |  |  |  |
| Total Points: |  |  | /3 | |
| **UVC** |  |  |  |  |
| Appropriate Decision based on clinical condition |  |  |  |  |
| Correct Insertion |  |  |  |  |
| Successful catheterization (<= 2 attempts Yes, >2 No) |  |  |  |  |
| Total Points: |  |  | /3 | |
|  |  |  |  |  |
| Total Points for evaluation: |  |  | /32 | |
|  |  |  |  |  |
|  |  |  |  |  |
|  |  |  |  |  |
|  |  |  |  |  |
|  |  |  |  |  |
| **Timing** | **In Seconds** |  |  |  |
| Time to first adequate BMV/PPV breath |  |  |  |  |
| Time to first chest compression |  |  |  |  |
| Time to successful Intubation |  |  |  |  |
| Duration of Intubation Attempts: |  |  |  |  |
| 1 |  |  |  |  |
| 2 |  |  |  |  |
| 3 |  |  |  |  |
| Time to first Epinephrine Dose |  |  |  |  |
| Time to vascular access |  |  |  |  |
| Time to first IV medication |  |  |  |  |
