# Supplementary material for: Rapid Cycle Deliberate Practice: Application to Neonatal Resuscitation
Source: MedEdPORTAL. 2017 Jan 30;13:10534. doi: 10.15766/mep_2374-8265.10534 (PMC6342166; doi:10.15766/mep_2374-8265.10534)
Supplement: Supplementary file 1 — A. Simulation Case.docx B. Critical Actions.docx C. Debriefing Materials.docx [file mep-13-10534-s001.zip › C. Debriefing Materials.docx]

**Tips for RCDP Instructors**

**Debriefing during RCDP**

*Stop and continue*

- Technical skills that are ongoing
- Ex. Compressions need to be faster

*Stop and backup 30 seconds*

- Skills that were just completed
- Ex. Coordination of pulse check
- Ex. Missed opportunity for shared mental model

*Stop and restart at beginning of round*

- Team heading in unexpected direction
- Ex. VT confused with PEA

**Debriefing Techniques**

Pick type of debriefing as appropriate for message

- Directive Feedback: Rate of compressions
- +/ Δ: Pulse Check coordination
- Advocacy and Inquiry: Repeated Errors, Unanticipated directions

**Praise List**

*Good communication:*

- Closed Loop Communication
- Shared Mental Models
- Mutual Respect

*Good Techniques*

- High quality Chest Compressions
- High quality ventilations (Goldilocks, not too fast, not too slow)
- Coordinated Pulse Check

*Action-linked phrases (PALS)*

- Patient is not breathing, I will/you will give breaths
- No pulse, starting compressions
- VF/VT preparing to defibrillate
- We have a rhythm change, checking pulse/ check pulse.

*Action-linked phrases (NRP)*

- "Patient has HR < 100 (or gasping or apnea)-- I'm starting respirations" (said and done by airway)
- After 30 seconds, “Patient has HR < 60—I am adjusting ventilation”
- After 30 seconds, “Patient still has HR < 60- I am starting compressions”

**Example of feedback:**

“I noticed that you have not checked a Heart Rate in over a minute, per the NRP guidelines the heart rate should be checked 30 seconds are starting effective ventilation, let’s reset for one minute ago when you started PPV, this time pausing to check the heart rate 30 seconds in.”
